# Factors associated with oral frailty in elderly stroke patients in Eastern China

**DOI:** 10.1590/1980-220X-REEUSP-2025-0020en

**Published:** 2025-12-08

**Authors:** Zhao Jiayue, Yujia Yujia, Xia Dandan, Zhou Minmin, Zhang Qing, Xu Lu, Sun Haimin, Xu Hongmei

**Affiliations:** 1 Binzhou Medical University Hospital, Binzhou, Shandong Province, China.; 2 School of Nursing, Binzhou Medical University, Binzhou, Shandong Province, China.; 3 Qilu Medical University, Zibo, Shandong Province, China.; 4 Binzhou Medical University, Science and Technology Department, Yantai, Shandong Province, China.

**Keywords:** Aged, Stroke, Oral Health, Nursing Care, Idosos, Acidente Vascular Cerebral, Saúde bucal, Cuidados de enfermagem

## Abstract

**Objective:**

To investigate the current status of oral frailty in elderly stroke patients in East China and analyze its associated factors, in order to provide a reference for the implementation of targeted interventions.

**Method:**

From June to September 2024, a convenience sample of 300 elderly stroke patients from two tertiary hospitals in Binzhou, Shandong underwent assessment with the General Information Questionnaire, Oral Frailty Index-8 (OFI-8), Oral Health Assessment Tool, Perceived Social Support Scale, and Geriatric Self-Efficacy in Oral Health Scale. Data were subjected to descriptive and inferential analysis.

**Results:**

The prevalence of oral frailty in elderly stroke patients was 47.0%, and age, smoking history, past history of stroke, number of teeth, subjective masticatory difficulty, oral health score, and perceived social support scale were the factors influencing oral frailty in elderly stroke patients (*P* < 0.05).

**Conclusion:**

In Eastern China, where nearly half of elderly stroke patients exhibit oral frailty, caregivers should prioritize routine OFI-8 screening and implement individualized interventions during stroke rehabilitation to break the oral frailty-stroke recurrence cycle.

## RESUMO

**Objetivo:**

Investigar a situação atual da fragilidade oral em pacientes idosos com AVC na China Oriental e analisar os fatores associados, a fim de fornecer uma referência para a implementação de intervenções direcionadas.

**Método:**

De junho a setembro de 2024, uma amostra de conveniência de 300 pacientes idosos com AVC de dois hospitais terciários em Binzhou, Shandong, foi avaliada com o Questionário de Informações Gerais, Índice de Fragilidade Oral-8 (OFI-8), Ferramenta de Avaliação da Saúde Oral, Escala de Apoio Social Percebido e Escala de Autoeficácia Geriátrica em Saúde Oral. Os dados foram submetidos a análise descritiva e inferencial.

**Resultados:**

A prevalência de fragilidade oral em pacientes idosos com AVC foi de 47,0%, e idade, histórico de tabagismo, histórico anterior de AVC, número de dentes, dificuldade mastigatória subjetiva, pontuação de saúde bucal e escala de apoio social percebido foram os fatores que influenciaram a fragilidade oral em pacientes idosos com AVC (*P* < 0,05).

**Conclusão:**

No leste da China, onde quase metade dos pacientes idosos com AVC apresentam fragilidade oral, os cuidadores devem priorizar a triagem de rotina OFI-8 e implementar intervenções individualizadas durante a reabilitação do AVC para quebrar o ciclo de fragilidade oral-recorrência do AVC.

## INTRODUCTION

Global population aging has amplified stroke prevalence and geriatric syndromes^(1)^. Oral frailty was originally a concept developed in Japan to describe the transitional decline of oral status from health to dysfunction with age, including oral problems such as tooth loss and reduced chewing function associated with swallowing, the effect of which is an overall deterioration of physical and mental function^(2)^. Its prevalence varies geographically: 16% in Japan’s prevention-focused communities, 29.5% among community-dwelling older adults in China, and 17.7% in Finnish care facilities, reflecting disparities in healthcare systems and prevention strategies^(3,4,5)^.

In stroke patients, dysphagia and cognitive, and motor impairments compromise oral hygiene practices, fueling a vicious cycle of biofilm buildup, chronic inflammation, and functional decline. This cascade is amplified in Eastern China’s urban-rural divide, where cutting-edge hospitals coexist with under-resourced clinics, and dietary shifts toward processed, high-salt foods exacerbate oral frailty^(6, 7)^.

Building upon these regional disparities and clinical challenges, the objective of this study was to investigate the prevalence of oral frailty and identify associated factors among elderly stroke patients in Eastern China, using the validated Oral Frailty Index-8 assessment tool. This investigation provides critical evidence to inform targeted nursing interventions across varied healthcare settings.

## METHOD

### Study Design

Cross-sectional quantitative study.

### Setting and Sample

From June to September 2024, 315 elderly stroke patients hospitalized in 2 tertiary general hospitals in Binzhou City, Shandong Province, East China, were selected by convenience sampling method as the study subjects. The inclusion criteria were as follows: ①Age ≥ 60 years; ②Meets diagnostic criteria for stroke as outlined in the American Heart Association/American Stroke Association guidelines^(8)^. ③Time of admission ≥ 24h; ④Barthel Index ≥ 60 (ensures patients maintain basic self-care abilities, such as tooth brushing and independent feeding, and can complete oral assessments either independently or with minimal assistance to participate in the study)^(9)^; ⑤Hospitalized patients who are conscious and able to communicate normally, with a Montreal Cognitive Assessment (MoCA) score ≥ 26^(10)^; ⑥The patients themselves and their families gave informed consent and volunteered to participate in the study. The exclusion criteria are as follows: ①With severe organ dysfunction. The sample size was calculated using G*Power 3.1 software with a medium effect size *f* ^2^ = 0.15, α = 0.05, power (1-β) = 0.9, and 27 predictors, resulting in a required sample size of 240 after accounting for a 10% non-response rate. This study ultimately included 300 participants to ensure robust statistical power and address potential data incompleteness.

## INSTRUMENT

### General Information Survey Form

The survey tool was a self-administered questionnaire developed through a synthesis of existing literature. It consisted of two sections: demographic characteristics and clinical profiles. Demographic characteristics included gender (male/female), age (reported as a continuous variable with median and interquartile range), employment status (unemployed/retired), education level (elementary school or below, junior high school, secondary school or above), body mass index (BMI) categorized as underweight (< 18.5 kg/m^2^), normal (18.5–23.9 kg/m^2^), or overweight/obese (≥ 24 kg/m^2^) based on WHO Asian criteria, household registration type (rural/urban/municipal district), marital status (married/other [unmarried, divorced, widowed]), living arrangement (living alone/cohabiting with family or spouse), monthly household income per capita (< ¥3,000, ¥3,000–¥5,000, >¥5,000), medical payment method (employee health insurance/resident health insurance/self-paid), smoking history (yes/no), and alcohol use history (yes/no). The clinical profiles section assessed chronic conditions (hypertension, diabetes, respiratory disease, cancer history, and prior cerebral infarction; all coded as yes/no), the number of concurrent chronic conditions (0, 1, or ≥ 2), polypharmacy (defined as concurrent use of ≥ 5 medications; yes/no), dental status (natural teeth categorized as < 10, 10–20, or > 20; denture count reported as a continuous variable with median and interquartile range), self-reported xerostomia (yes/no), and subjective masticatory difficulty (yes/no).

### Oral Frailty Index-8 (OFI-8)

The scale was used to assess the degree of oral frailty in patients^(11)^. It consists of 5 dimensions, including whether or not to use a denture, swallowing ability, chewing ability, oral health-related behaviors, and social participation, with a total of 8 entries. Scores ranged from 0 to 11 on the scale, with a cutoff of ≥ 4 defining oral frailty (dichotomized as present/absent). Higher scores reflected worse oral health status in subsequent analyses. The scale, as measured in China, showed Cronbach’s α 0.949 and content validity 0.934.

### The Oral Health Assessment Tool (OHAT)

The scale was revised by Australian scholars Chalmers et al. in 2005 based on the Simple Oral Health Checklist^(12)^. It aims to assess the oral health status of the elderly, covering 8 oral-related areas with a total score of 16 points. < 3 points indicate oral health, ≥ 3 points indicate poor oral health, and the higher the score, the worse the oral health status. The scale demonstrated good internal consistency in China, with a Cronbach’s α of 0.710 and retest reliability of 0.811, and is suitable for stroke patients.

### Geriatric Self-Efficacy In Oral Health Scale (GSEOH)

The scale was compiled by Ohara et al. in 2017 and consists of three dimensions: oral function, oral hygiene habits, and oral clinic habits^(13)^. It contains 20 items with a total score of 80 points. The higher the score, the stronger the self-efficacy of the elderly in terms of oral health (data are expressed as median (P25, P75)). The Chinese version demonstrated good reliability with Cronbach’s α 0.913 and retest reliability of 0.743.

### Perceived Social Support Scale (PSSS)

This study employed the 12-item Chinese version of the Perceived Social Support Scale, which was translated and culturally adapted by Jiang Qianjin^(14)^, originally developed by Zimet et al. to measure an individual’s self-perceived multidimensional social support^(15)^. It includes three dimensions: family support, friend support, and other support. The Likert 7-point scoring method is used, with 12 points to 36 points as a low support level, 37 points to 60 points as an intermediate support level, and 61 points to 84 points as a high support level. The culturally adapted scale applied in China demonstrated a Cronbach’s α of 0.840.

### Data Collection

There was a unified training of investigators by the research group. The investigator gave training using a unified guide to explain to the survey respondents to fill out the method, after getting consent to fill out the questionnaire on their own. For those who were not able to fill in the survey, the researcher with no hint of explanation, patiently dictate the content of the questionnaire, to be answered by the respondents to fill in for the sake of the respondents. If the recovered questionnaires were regular answers, the same option or incomplete answers, they were regarded as invalid questionnaires and were eliminated. A total of 315 questionnaires were distributed in this survey, and 300 valid questionnaires were recovered, with an effective recovery rate of 95.2%.

### Data Analysis

Statistical analyses were performed using SPSS 26.0: t-test for continuous variables with normal distribution. Mann-Whitney test for variables without normal distribution. Chi-square for categorical variables. Variables showing statistical significance in univariate analyses were subsequently entered into binary logistic regression analysis using a forward stepwise selection method to identify independent factors associated with oral frailty. Receiver Operating Characteristic (ROC) curve analysis was conducted in R software (v4.2.1), with the area under the curve (AUC) quantifying the model’s predictive performance. Statistical significance was set at *P* < 0.05.

### Ethics Statement

This study was conducted in accordance with the Declaration of Helsinki and was approved by the Ethics Committee of Binzhou Medical University (Approval No. 2024-L031). Written informed consent was obtained from all participants after they were informed about the purpose and significance of the research.

## RESULTS

### Current Status of Oral Frailty in Elderly Stroke Patients

A total of 300 elderly stroke patients participated in this study, including 141 (47.0%) in the oral frailty group and 159 (53.0%) in the non-oral frailty group; 135 (45.0%) with a previous history of cerebral infarction and 165 (55.0%) without a history of cerebral infarction; and the number of teeth was < 10 in 87 cases (29.0%), 10–20 in 62 cases (20.7%), and > 20 teeth in 151 cases (50.3%), see Table 1 for details.

**Table 1:** Univariate analysis of oral frailty in elderly stroke patients (n = 300). East China, China, 2024.

Variable	OFI Group	Non-OFI group	Z*/χ^2^	*P*
Gender			1.490	0.222
Male	78(55.3)	99(62.3)		
Female	63(44.7)	60(37.7)		
Age M(P25,P75)	72(63,74)	63(62,66)	−7.600*	<0.001
Working conditions			52.777	<0.001
Unemployed	124(87.9)	77(48.4)		
Retired	17(12.1)	82(51.6)		
Educational scale			52.974	<0.001
Primary Education	96(68.1)	46(28.9)		
Middle school	37(26.2)	65(40.9)		
Junior college and above	8(5.7)	48(30.2)		
BMI			3.636	0.162
<18.5	11(7.8)	6(3.8)		
≥ 18.5, <24	53(37.6)	73(45.9)		
≥ 24	77(54.6)	80(50.3)		
Permanent Address			33.742	<0.001
Rural	109(77.3)	71(44.7)		
Town	24(17.0)	59(37.1)		
City center	8(5.7)	29(18.2)		
Marital Status			4.419	0.036
Married	122(86.5)	149(93.7)		
Others (unmarried, divorced, widowed)	19(13.5)	10(6.3)		
Residence status			3.737	0.053
Living alone	13(9.2)	6(3.8)		
Living with spouse	128(90.8)	153(96.2)		
Monthly per capita family income			2.011	0.366
< 3000 RMB	29(20.6)	23(14.5)		
3000 RMB−5000 RMB	62(44.0)	73(45.9)		
>5000 RMB	50(35.5)	63(39.6)		
Payment of medical expenses			44.711	<0.001
Employee medical insurance	17(12.1)	76(47.8)		
Residents’ medical insurance	123(87.2)	82(51.6)		
Self−financed	1(0.7)	1(0.6)		
Smoking history			7.079	0.008
Cigarette smoking	59(41.8)	91(57.2)		
Non−smoking	82(58.2)	68(42.8)		
Drinking history			0.038	0.846
Drinking wine	99(70.2)	110(69.2)		
No alcohol	42(29.8)	49(30.8)		
Hypertension			7.105	0.008
No	30(21.3)	56(35.2)		
Yes	111(78.7)	103(64.8)		
Cardiovascular Disease			1.963	0.161
No	114(80.9)	138(86.8)		
Yes	27(19.1)	21(13.2)		
Diabetes			8.673	0.003
No	83(58.9)	119(74.8)		
Yes	58(41.1)	40(25.2)		
Respiratory Disease			0.034	0.855
No	136(96.5)	155(97.5)		
Yes	5(3.5)	4(2.5)		
Cancer			5.410	0.020
No	126(89.4)	153(96.2)		
Yes	15(10.6)	6(3.8)		
Past History of Stroke			122.245	<0.001
No	30(21.3)	135(84.9)		
Yes	111(78.7)	24(15.1)		
Number of chronic diseases			28.075	<0.001
none	10(7.1)	36(22.6)		
1 type	35(24.8)	61(38.4)		
>1 type	96(68.1)	62(39.0)		
Whether polypharmacy (≥ 5 drugs)			11.360	0.001
No	10(7.1)	33(20.8)		
Yes	131(92.9)	126(79.2)		
Number of teeth			142.208	<0.001
Less than 10	82(58.2)	5(3.1)		
10 to 20	36(25.5)	26(16.4)		
More than 20	23(16.3)	128(80.5)		
Number of dentures M(P25,P75)	14(4,24)	0(0,1)	−13.120*	<0.001
Dry mouth			32.855	<0.001
No	42(29.8)	100(62.9)		
Yes	99(70.2)	59(37.1)		
Subjective masticatory difficulty			153.205	<0.001
No	17(12.1)	133(83.6)		
Yes	124(87.9)	26(16.4)		
OHAT			130.069	<0.001
No	113(80.1)	23(14.5)		
Yes	28(19.9)	136(85.5)		
Perceived Social Support scale			33.551	<0.001
Low	68(48.2)	51(32.1)		
Medium	56(39.7)	41(25.8)		
High	17(12.1)	67(42.1)		
GSEOH score M(P25,P75)	46(43,49)	55(49,57)	−9.341*	<0.001

### A Single Factor Analysis of Oral Frailty Affecting Elderly Stroke Patients

The OFI-8 score of the respondents in this study was (4.87 ± 2.63), in which the prevalence of oral frailty was 47.0%; the results of univariate analysis showed that age, work conditions, education scale, permanent address, marital status, Payment of medical expenses, history of smoking, history of hypertension, history of diabetes mellitus, history of cancer, history of cerebral infarcts, number of chronic illnesses, multiple medication use, number of teeth, number of dentures, dry mouth condition, subjective masticatory difficulty, oral health status, perceived social support scale, and GSEOH score were the associated factors of oral frailty in elderly stroke patients, and the difference was statistically significant(*P* < 0.05), as shown in Table 1.

### Multifactorial Analysis of Oral Frailty in Elderly Stroke Patients

Logistic regression revealed significant associations between oral frailty and several factors, including advanced age, smoking history, prior stroke, reduced natural teeth, subjective masticatory difficulty, poor oral health, and low social support (detailed results in Table 2). The strongest predictors were subjective masticatory difficulty (OR = 4.743, 95% CI: 1.343–16.751) and smoking history (OR = 3.589, 95% CI: 1.291–9.974), followed by age (OR = 1.136, 95% CI: 1.020–1.265).

**Table 2:** Logistic regression results of oral frailty in elderly stroke patients. East China, China, 2024.

Variable	β	SE	Waldχ^2^	*P*	OR	95%CI
Age	0.128	0.055	5.410	0.020	1.136	1.020–1.265
Smoking history	1.278	0.521	6.005	0.014	3.589	1.291–9.974
Past History of Stroke	1.041	0.526	3.915	0.048	2.833	1.010–7.950
Number of teeth (Less than 10)			9.375	0.009		
10 to 20	−1.800	0.804	5.013	0.025	0.165	0.034–0.799
More than 20	−2.899	0.947	9.372	0.002	0.055	0.009–0.352
Subjective masticatory difficulty	1.557	0.644	5.845	0.016	4.743	1.343–16.751
OHAT	−1.238	0.552	5.040	0.025	0.290	0.098–0.855
Perceived Social Support scale(Low)			12.226	0.002		
Medium	−2.289	0.724	9.991	0.002	0.101	0.025–0.419
High	−1.685	0.705	5.715	0.017	0.185	0.047–0.738

### Logistic Regression Analysis of Oral Frailty in Elderly Stroke Patients

**Figure 1:**
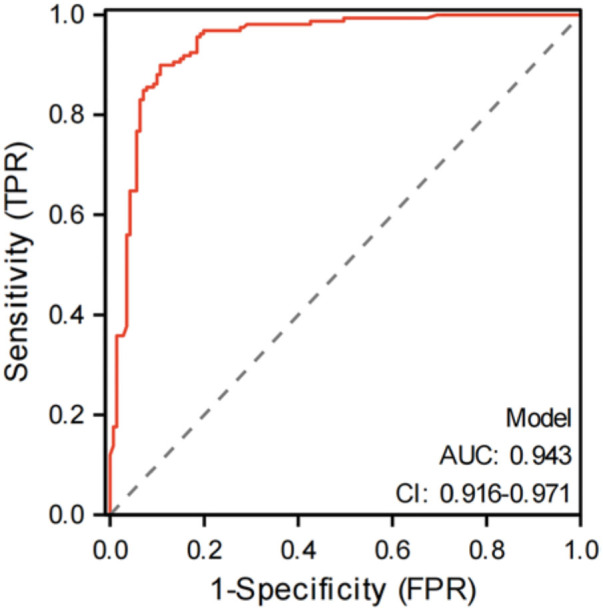
Logistic regression model.

The AUC of the logistic regression model for oral frailty in elderly stroke patients was 0.943[95%CI(0.916, 0.971)], as shown in Figure 1.

## DISCUSSION

### Higher Incidence of Oral Frailty in Elderly Stroke Patients

The prevalence among elderly stroke patients in East China reached 47.0%, substantially exceeding the average rate among China’s community-dwelling elderly population (40.2%). Notably, this rate also markedly surpassed records from Japan and Finland. This disparity likely stems from: ①Stroke-specific vulnerabilities: Motor/cognitive impairments and dysphagia synergistically compromise oral hygiene; ②Methodological divergence: Our OFI-8 tool’s functional domains capture stroke sequelae better than Finland’s clinical indicator-focused checklist; ③Regional healthcare constraints: China’s urban-rural resource imbalance and prevention-poor insurance systems delay oral frailty interventions. Given nurses’ pivotal role in China’s healthcare system, training should emphasize identifying stroke-related oral issues, incorporating routine assessments and interventions (e.g., assisted brushing), guiding patient/family education on oral self-care, and partnering with dental teams to establish interdisciplinary protocols—optimizing care coordination to enhance outcomes and reduce stroke recurrence risks.

### Factors Influencing Oral Frailty in Elderly Stroke Patients

Binary logistic regression analysis revealed that having >20 natural teeth, better oral health (OHAT score < 3), and high social support (PSSS score ≥ 61) were protective factors against oral frailty in elderly stroke patients, whereas older age, smoking, prior stroke, and masticatory difficulty were independently associated with increased risk.

### Advanced Age

The results of this study indicate that increasing age is associated with a greater risk of oral fragility in patients with stroke(*P* < 0.05), which is consistent with the results of previous studies^(16)^. The prevalence of oral frailty increases with age, largely due to degenerative changes in oral function and pathology. Biologically, aging reduces periodontal stem cell regeneration and alkaline phosphatase activity, disrupting the osteogenic-osteolytic balance of the alveolar bone. Functionally, decreased salivary secretion weakens the oral immune barrier, accelerating periodontal atrophy and loss of gingival integrity. Pathologically, degenerative changes and microbiota dysregulation jointly contribute to conditions such as periodontitis and dental caries. These factors create a positive feedback loop, ultimately driving the progression of oral frailty^(17)^. In addition, elderly stroke patients have limited cognitive and physical mobility, fall into partial or complete incapacitation, and have a reduced ability to take care of themselves leading to poor oral health, which in turn increases the risk of oral frailty. Studies have shown that the more informed patients are about poor oral health-related outcomes, the more proactive they are in maintaining their oral health^(18)^. To improve oral health in elderly stroke patients, nursing staff should actively promote oral health education and provide hygiene guidance. Strengthening patients’ awareness and encouraging proper oral hygiene habits can help maintain better oral health outcomes.

### Smoking History

The results of this study showed a statistically significant association between smoking history and oral frailty in elderly stroke patients(*P* < 0.05). Elderly stroke patients who smoke have a high risk of developing oral frailty, which is consistent with the results of previous studies^(19)^. Studies have demonstrated that smoking disrupts oral microbiota equilibrium, promoting dysbiosis linked to caries, periodontal disease, and oral ulceration^(20)^. In addition, reduced oral microbial diversity is associated with neurological, cardiovascular, respiratory, and cancer health problems. Smoking significantly increases the risk of stroke by causing hardening and narrowing of the blood vessels and damage to the lining of the blood vessels, reducing blood supply to the brain. Although the dangers of smoking are widely known, 50%of the elderly stroke patients in this study still smoked; therefore, healthcare professionals should actively persuade patients to quit smoking and provide regular oral health checkups for early detection and treatment of oral problems caused by smoking. At the same time, the nurse team should conduct regular smoking cessation education and oral health education, and provide patients with personalized support such as smoking cessation counseling, behavioral interventions, oral hygiene instruction, and medication to reduce the risk of oral frailty and stroke.

### Past History of Stroke

The results of this study showed that history of previous cerebral infarction was an independent risk factor for oral frailty in elderly stroke patients (*P* < 0.05), concurrent with the findings reported by Hironaka et al.^(21)^. Most stroke patients suffer from varying degrees of oral problems. Strokes may affect swallowing function through brain damage, which triggers a wide range of symptoms related to oral hypoplasia. Post-stroke patients often face dental anxiety and dysphagia, problems that often lead to more severe oral frailty^(22)^. Not only does oral frailty make patients more susceptible to oral infections, but it may also raise the risk of stroke recurrence. In addition, elderly stroke patients often wear dentures due to more missing teeth, which, along with the side effects of stroke medications, further increases the risk of developing oral diseases^(23)^. Numerous studies have shown that older adults with chronic diseases such as cognitive frailty, Alzheimer’s disease, cardiovascular disease, stroke, and diabetes are more likely to experience oral frailty. Post-stroke patients are often accompanied by neurological dysfunction, cognitive impairment, dysphagia, and decreased mobility, resulting in decreased oral salivation and food retention, inability to clean up oral hygiene in a timely manner, and low scales of oral health self-efficacy, which can affect oral function. In addition, studies have shown that oral inflammation such as tooth loss and periodontal disease may cause oral bacterial infections which may directly promote atherosclerosis and thrombosis, thus adversely affecting the cardiovascular system^(24)^. Therefore, for elderly patients with a history of stroke, their oral function management should be strengthened, oral hygiene should be emphasized, and oral health awareness should be improved. Standardized tools are used in a multidisciplinary team collaboration, in order to assess oral muscle strength, tongue pressure, and chewing efficiency, and compensatory swallowing, as well as strategy training is carried out, such as multiple swallowing and head position adjustment, to provide patients with comprehensive and personalized oral management.

### Number of Teeth, Subjective Masticatory Difficulty, Oral Health Status

The results of this study showed that elderly stroke patients with < 20 teeth, subjective chewing difficulties, and poor oral health were more likely to develop oral frailty (*P* < 0.05), consistent with the findings of Tanaka et al.^(25)^. The number of teeth, as a measure of overall health, reflects the accumulation of oral problems^(18)^. Tooth loss can directly cause chewing difficulties, which not only affects nutritional intake and quality of life but also increases the risk of death. One study showed that for every 10 teeth lost, the risk of death increased by 15%, 33%and 57%respectively^(26)^. In addition, a low number of teeth and reduced chewing ability are associated with cognitive decline, whereas about half of stroke patients experience varying degrees of cognitive impairment. Elderly people with impaired cognitive function may have inadequate knowledge of oral health, leading to reduced compliance with oral hygiene habits and self-care, which can increase the scale of oral inflammation and raise the risk of oral frailty. It has been shown that as patients know more about oral health related adverse health outcomes, the more active they are in maintaining their oral health^(27)^. Poor oral health mainly includes oral dryness, missing teeth, oral mucosal disease, periodontal disease or dental caries. Decreased salivary secretion caused by oral dryness increases the risk of candida albicans infection by weakening the inhibitory effects of salivary glycoproteins and antimicrobial proteins. Therefore, insufficient saliva and poor oral hygiene habits may cause oral microbial dysbiosis triggering oral infections, which in turn leads to oral frailty. In addition, elderly stroke patients with fewer teeth are more likely to have difficulty chewing, which is associated with weakened masticatory and perioral muscle strength and reduced chewing efficiency, and this decline in chewing function may exacerbate cognitive impairment through a variety of pathways such as reducing signaling, decreasing cerebral blood flow, and triggering chronic oxidative stress damage to the hippocampus and vertebral cell reduction^(28)^. In summary, elderly stroke patients with poor oral health are more prone to oral frailty, which not only affects masticatory function, but may also aggravate cognitive impairment. Therefore, as healthcare professionals, we should strengthen oral health education for patients, raise awareness of the consequences of poor oral health, and provide professional oral hygiene guidance. At the same time, for elderly stroke patients, special attention should be paid to patients’ masticatory function and oral health, and the risk of oral frailty and cognitive impairment should be minimized through a comprehensive management strategy including oral rehabilitation training and appropriate dietary adjustments. Oral health is extremely critical to maintaining good overall health, and emphasizing and maintaining oral health is essential to prevent oral frailty and improve the overall health of stroke patients.

### Perceived Social Support Scale

This study identified perceived social support as an independent protective factor against oral frailty in elderly stroke patients (*P* < 0.05), aligning with prior evidence of its negative association with oral frailty risk^(29)^. Stroke-induced motor/cognitive impairments impair social functioning, reducing communication opportunities that drive declines in tongue pressure, chewing capacity, and oral muscle activity^(21)^. Oral functional decline (e.g., tooth loss) may further exacerbate social anxiety and withdrawal^(30)^, fostering loneliness and depression that disrupt oral hygiene behaviors. This creates a self-reinforcing ‘psycho-social-physical’ cycle. Nurse-led multidimensional prevention strategies should integrate bedside mental health screening, tailored cognitive-behavioral coaching for oral hygiene adherence, and community-based social engagement programs, while collaborating with dental teams to standardize evidence-based oral care protocols for this high-risk population.

## CONCLUSION

The high prevalence of oral frailty in elderly stroke patients in East China (47.0%), which was associated with factors related to advanced age, history of smoking, previous history of stroke, low number of teeth, difficulty in chewing, poor oral condition, and low level of social support, suggests an urgent need for systematic oral health management. Key recommendations include: 1) implementing nurse-led OFI-8 screenings during hospitalization with tailored strategies such as adaptive tools for hemiplegia and saliva substitutes for dry mouth; 2) establishing interdisciplinary neurology-dental teams to standardize post-stroke care pathways. Future studies must prioritize validating culturally adapted OFI-8 versions in underrepresented populations such as rural and nursing home residents, while public health policies should mandate oral assessments in rehabilitation guidelines and subsidize adaptive devices to address urban-rural disparities. These steps target universal and region-specific barriers, promoting equitable global implementation.

This study has limitations: the research employed a cross-sectional design, which cannot determine causality among the study variables, and single-region sampling limits generalizability. Future multi-center longitudinal studies across diverse regions, particularly targeting rural and nursing home populations, are essential to clarify oral frailty progression and optimize interventions.

## Data Availability

The entire dataset supporting the results of this study was published in the article itself.
