# Supplementary material for: Factors associated with oral frailty in elderly stroke patients in Eastern China
Source: Rev Esc Enferm USP. 2025 Dec 8;59:e20250020. doi: 10.1590/1980-220X-REEUSP-2025-0020en (PMC12685079; doi:10.1590/1980-220X-REEUSP-2025-0020en)
Supplement: Translation

# Translation: Factores asociados con la fragilidad oral en pacientes ancianos con accidente cerebrovascular en el este de China

**DOI:** 10.1590/1980-220X-REEUSP-2025-0020es

**Authors:** Zhao Jiayue, Yujia Yujia, Xia Dandan, Zhou Minmin, Zhang Qing, Xu Lu, Sun Haimin, Xu Hongmei

**Affiliations:** 1 Binzhou Medical University Hospital, Binzhou, Shandong Province, China.; 2 School of Nursing, Binzhou Medical University, Binzhou, Shandong Province, China.; 3 Qilu Medical University, Zibo, Shandong Province, China.; 4 Binzhou Medical University, Science and Technology Department, Yantai, Shandong Province, China.

**Keywords:** Anciano, Accidente cerebrovascular, Salud Bucal, Atención de Enfermería

## Abstract

**Objetivo:**

Investigar la situación actual de la fragilidad oral en pacientes ancianos con accidente cerebrovascular en el este de China y analizar los factores asociados, con el fin de proporcionar una referencia para la implementación de intervenciones específicas.

**Método:**

Entre junio y septiembre de 2024, se evaluó a una muestra de conveniencia de 300 pacientes ancianos con accidente cerebrovascular de dos hospitales terciarios de Binzhou, Shandong, mediante el Cuestionario de Información General, el Índice de Fragilidad Oral-8 (OFI-8), la Herramienta de Evaluación de la Salud Oral, la Escala de Apoyo Social Percibido y la Escala de Autoeficacia Geriátrica en Salud Oral. Los datos se sometieron a un análisis descriptivo y inferencial.

**Resultados:**

La prevalencia de la fragilidad oral en pacientes ancianos con accidente cerebrovascular fue del 47,0%, y la edad, el historial de tabaquismo, los antecedentes de accidente cerebrovascular, el número de dientes, la dificultad masticatoria subjetiva, la puntuación de salud bucodental y la escala de apoyo social percibido fueron los factores que influyeron en la fragilidad oral en pacientes ancianos con accidente cerebrovascular (P < 0,05).

**Conclusión:**

En el este de China, donde casi la mitad de los pacientes ancianos con accidente cerebrovascular presentan fragilidad oral, los cuidadores deben dar prioridad a las pruebas de detección rutinarias con la OFI-8 y aplicar intervenciones individualizadas durante la rehabilitación del accidente cerebrovascular para romper el ciclo de fragilidad oral-recurrencia del accidente cerebrovascular.

## INTRODUCCIÓN

El envejecimiento de la población mundial ha amplificado la prevalencia de los accidentes cerebrovasculares y los síndromes geriátricos^(1)^. La fragilidad oral fue originalmente un concepto desarrollado en Japón para describir el deterioro transitorio del estado bucal, desde la salud hasta la disfunción con la edad, incluyendo problemas bucales como la pérdida de dientes y la reducción de la función masticatoria asociada a la deglución, cuyo efecto es un deterioro general de la función física y mental^(2)^. Su prevalencia varía según la zona geográfica: 16% en las comunidades japonesas centradas en la prevención, 29,5% entre los adultos mayores que viven en comunidades en China y 17,7% en los centros de atención finlandeses, lo que refleja las disparidades en los sistemas de salud y las estrategias de prevención^(3–5)^.

En los pacientes con accidente cerebrovascular, la disfagia y las deficiencias cognitivas y motoras comprometen las prácticas de higiene bucal, lo que alimenta un círculo vicioso de acumulación de biopelícula, inflamación crónica y deterioro funcional. Esta cascada se amplifica en la división entre las zonas urbanas y rurales del este de China, donde coexisten hospitales de vanguardia con clínicas con pocos recursos, y los cambios en la dieta hacia alimentos procesados y con alto contenido de sal exacerban la fragilidad bucal^(6, 7)^.

Partiendo de estas disparidades regionales y retos clínicos, el objetivo de este estudio fue investigar la prevalencia de la fragilidad bucal e identificar los factores asociados entre los pacientes ancianos con accidente cerebrovascular en el este de China, utilizando la herramienta de evaluación validada Oral Frailty Index-8. Esta investigación proporciona pruebas fundamentales para informar sobre intervenciones de enfermería específicas en diversos entornos sanitarios.

## MÉTODO

### Diseño Del Estudio

Estudio cuantitativo transversal.

### Entorno y Muestra

Entre junio y septiembre de 2024, se seleccionó como sujetos del estudio a 315 pacientes ancianos con accidente cerebrovascular hospitalizados en dos hospitales generales terciarios de la ciudad de Binzhou, provincia de Shandong, este de China, mediante un método de muestreo por conveniencia. Los criterios de inclusión fueron los siguientes: ①Edad ≥ 60 años; ②Cumplimiento de los criterios de diagnóstico de accidente cerebrovascular descritos en las directrices de la American Heart Association/American Stroke Association^(8)^. ③Tiempo de ingreso ≥ 24 h; ④Índice de Barthel ≥ 60 (garantiza que los pacientes mantengan habilidades básicas de autocuidado, como cepillarse los dientes y alimentarse de forma independiente, y que puedan completar evaluaciones orales de forma independiente o con una ayuda mínima para participar en el estudio)^(9)^; ⑤Pacientes hospitalizados que estén conscientes y puedan comunicarse con normalidad, con una puntuación en la Evaluación Cognitiva de Montreal (MoCA) ≥ 26^(10)^; ⑥Los propios pacientes y sus familias dieron su consentimiento informado y se ofrecieron voluntariamente para participar en el estudio. Los criterios de exclusión son los siguientes: ①Con disfunción orgánica grave. El tamaño de la muestra se calculó utilizando el software G*Power 3.1 con un tamaño del efecto medio *f* ^2^ = 0,15, α = 0,05, potencia (1-β) = 0,9 y 27 predictores, lo que dio como resultado un tamaño de muestra requerido de 240 después de tener en cuenta una tasa de falta de respuesta del 10%. Este estudio incluyó finalmente a 300 participantes para garantizar una potencia estadística sólida y abordar la posible incompletitud de los datos.

## INSTRUMENTO

### Formulario de Encuesta de Información General

La herramienta de encuesta fue un cuestionario autoadministrado desarrollado a partir de una síntesis de la bibliografía existente. Constaba de dos secciones: características demográficas y perfiles clínicos. Las características demográficas incluían el sexo (masculino/femenino), la edad (informada como una variable continua con mediana y rango intercuartílico), la situación laboral (desempleado/jubilado), el nivel educativo (escuela primaria o inferior, escuela secundaria, escuela secundaria o superior), el índice de masa corporal (IMC) categorizado como bajo peso (< 18.5 kg/m^2^), normal (18.5–23.9 kg/m^2^) o sobrepeso/obesidad (≥ 24 kg/m^2^) según los criterios asiáticos de la OMS, tipo de registro de hogar (rural/urbano/distrito municipal), estado civil (casado/otro [soltero, divorciado, viudo]), situación de convivencia (vive solo/convive con su familia o cónyuge), ingresos mensuales per cápita del hogar (< ¥3000, ¥3000–5000, > ¥5000), método de pago de los gastos médicos (seguro médico para empleados/seguro médico para residentes/pago por cuenta propia), historial de tabaquismo (sí/no) e historial de consumo de alcohol (sí/no). En la sección de perfiles clínicos se evaluaron las enfermedades crónicas (hipertensión, diabetes, enfermedades respiratorias, antecedentes de cáncer y antecedentes de infarto cerebral; todas codificadas como sí/no), el número de enfermedades crónicas concurrentes (0, 1 o ≥ 2), la polifarmacia (definida como el uso simultáneo de ≥ 5 medicamentos; sí/no), estado dental (dientes naturales clasificados como < 10, 10–20 o > 20; número de dentaduras postizas indicado como variable continua con mediana y rango intercuartílico), xerostomía autoinformada (sí/no) y dificultad masticatoria subjetiva (sí/no).

### Índice de Fragilidad Oral-8 (Oral Frailty Index-8 - OFI-8)

La escala se utilizó para evaluar el grado de fragilidad oral en los pacientes^(11)^. Consta de 5 dimensiones, entre las que se incluyen el uso o no de dentaduras postizas, la capacidad de deglución, la capacidad de masticación, los comportamientos relacionados con la salud bucodental y la participación social, con un total de 8 entradas. Las puntuaciones oscilaron entre 0 y 11 en la escala, con un límite de ≥ 4 que definía la fragilidad oral (dicotomizada como presente/ausente). Las puntuaciones más altas reflejaban un peor estado de salud bucal en los análisis posteriores. La escala, tal y como se midió en China, mostró un α de Cronbach de 0,949 y una validez de contenido de 0,934.

### Herramienta de Evaluación de La Salud Bucal (Oral Health Assessment Tool - OHAT)

La escala fue revisada por los académicos australianos Chalmers et al. en 2005 basándose en la lista de verificación simple de salud bucal^(12)^. Su objetivo es evaluar el estado de salud bucodental de las personas mayores, cubriendo ocho áreas relacionadas con la salud bucodental con una puntuación total de 16 puntos. Menos de tres puntos indican buena salud bucodental, tres puntos o más indican mala salud bucodental, y cuanto mayor es la puntuación, peor es el estado de salud bucodental. La escala demostró una buena consistencia interna en China, con un α de Cronbach de 0,710 y una fiabilidad de repetición de la prueba de 0,811, y es adecuada para pacientes con accidente cerebrovascular.

### Escala de Autoeficacia Geriátrica en Salud Bucodental (Geriatric Self-Efficacy in Oral Health Scale - GSEOH)

La escala fue compilada por Ohara et al. en 2017 y consta de tres dimensiones: función bucodental, hábitos de higiene bucodental y hábitos de clínica bucodental^(13)^. Contiene 20 ítems con una puntuación total de 80 puntos. Cuanto mayor es la puntuación, mayor es la autoeficacia de las personas mayores en términos de salud bucodental (los datos se expresan como mediana (P25, P75)). La versión china demostró una buena fiabilidad con un α de Cronbach de 0,913 y una fiabilidad de repetición de la prueba de 0,743.

### Escala de Apoyo Social Percibido (Perceived Social Support Scale - PSSS)

Este estudio empleó la versión china de 12 ítems de la Escala de apoyo social percibido, traducida y adaptada culturalmente por Jiang Qianjin^(14)^, desarrollada originalmente por Zimet et al. para medir el apoyo social multidimensional percibido por el individuo^(15)^. Incluye tres dimensiones: apoyo familiar, apoyo de amigos y otros apoyos. Se utiliza el método de puntuación Likert de 7 puntos, con 12 a 36 puntos como nivel de apoyo bajo, 37 a 60 puntos como nivel de apoyo intermedio y 61 a 84 puntos como nivel de apoyo alto. La escala adaptada culturalmente aplicada en China demostró un α de Cronbach de 0,840.

### Recolección de Datos

El grupo de investigación impartió una formación unificada a los investigadores. El investigador impartió la formación utilizando una guía unificada para explicar a los encuestados cómo rellenar el cuestionario, después de obtener su consentimiento para rellenarlo por su cuenta. A aquellos que no pudieron rellenar la encuesta, el investigador, sin dar ninguna explicación, les dictó pacientemente el contenido del cuestionario para que lo rellenaran en su nombre. Si los cuestionarios recuperados contenían respuestas irregulares, la misma opción o respuestas incompletas, se consideraban cuestionarios no válidos y se eliminaban. En esta encuesta se distribuyeron un total de 315 cuestionarios y se recuperaron 300 cuestionarios válidos, con una tasa de recuperación efectiva del 95,2%.

### Análisis de Datos

Los análisis estadísticos se realizaron con el programa SPSS 26.0: prueba t para variables continuas con distribución normal. Prueba de Mann-Whitney para variables sin distribución normal. Chi-cuadrado para variables categóricas. Las variables que mostraron significación estadística en los análisis univariantes se introdujeron posteriormente en un análisis de regresión logística binaria utilizando un método de selección por pasos hacia adelante para identificar los factores independientes asociados con la fragilidad oral. El análisis de la curva característica operativa del receptor (Receiver Operating Characteristic-ROC) se realizó en el software R (v4.2.1), cuantificando el rendimiento predictivo del modelo mediante el área bajo la curva (area under the curve-AUC). La significación estadística se fijó en *P* < 0,05.

### Declaración Ética

Este estudio se llevó a cabo de conformidad con la Declaración de Helsinki y fue aprobado por el Comité de Ética de la Universidad Médica de Binzhou (n.° de aprobación 2024-L031). Se obtuvo el consentimiento informado por escrito de todos los participantes después de informarles sobre el propósito y la importancia de la investigación.

## RESULTADOS

### Estado Actual de La Fragilidad Oral En Pacientes Ancianos Con Accidente Cerebrovascular

En este estudio participaron un total de 300 pacientes ancianos con accidente cerebrovascular, incluidos 141 (47,0%) en el grupo de fragilidad oral y 159 (53,0%) en el grupo sin fragilidad oral; 135 (45,0%) con antecedentes de infarto cerebral y 165 (55,0%) sin antecedentes de infarto cerebral; y el número de dientes era < 10 en 87 casos (29,0%), entre 10 y 20 en 62 casos (20,7%) y más de 20 dientes en 151 casos (50,3%). Véase la Tabla 1 para más detalles.

**Tabla 1:** Análisis univariante de la fragilidad oral en pacientes ancianos con accidente cerebrovascular (n = 300). Este de China, China, 2024.

Variable	Grupo OFI	Grupo no OFI	Z*/χ^2^	*P*
Género			1,490	0,222
Masculino	78 (55,3)	99 (62,3)		
Mujer	63 (44,7)	60 (37,7)		
Edad M (P25, P75)	72 (63,74)	63 (62,66)	−7,600*	<0,001
Condiciones laborales			52,777	< 0,001
Desempleado	124 (87,9)	77 (48,4)		
Jubilados	17 (12,1)	82 (51,6)		
Escala educativa			52,974	< 0,001
Educación primaria	96 (68,1)	46 (28,9)		
Secundaria	37 (26,2)	65 (40,9)		
Escuela técnica superior y superior	8 (5,7)	48 (30,2)		
IMC			3,636	0,162
< 18,5	11 (7,8)	6 (3,8)		
≥ 18,5, < 24	53 (37,6)	73 (45,9)		
≥ 24	77 (54,6)	80 (50,3)		
Dirección permanente			33,742	< 0,001
Rural	109 (77,3)	71 (44,7)		
Pueblo	24 (17,0)	59 (37,1)		
Centro de la ciudad	8 (5,7)	29 (18,2)		
Estado civil			4,419	0,036
Casado	122 (86,5)	149 (93,7)		
Otros (solteros, divorciados, viudos)	19 (13,5)	10 (6,3)		
Estatus de residencia			3,737	0,053
Vive solo	13 (9,2)	6 (3,8)		
Vive con su cónyuge	128 (90,8)	153 (96,2)		
Ingresos familiares mensuales per cápita			2,011	0,366
< 3000 RMB	29 (20,6)	23 (14,5)		
3000 RMB-5000 RMB	62 (44,0)	73 (45,9)		
> 5000 RMB	50 (35,5)	63 (39,6)		
Pago de gastos médicos			44,711	< 0,001
Seguro médico para empleados	17 (12,1)	76 (47,8)		
Seguro médico para residentes	123 (87,2)	82 (51,6)		
Autofinanciado	1 (0,7)	1 (0,6)		
Antecedentes de tabaquismo			7,079	0,008
Consumo de cigarrillos	59 (41,8)	91 (57,2)		
No fumador	82 (58,2)	68 (42,8)		
Antecedentes de consumo de alcohol			0,038	0,846
Consumo de vino	99 (70,2)	110 (69,2)		
Sin alcohol	42 (29,8)	49 (30,8)		
Hipertensión			7,105	0,008
No	30 (21,3)	56 (35,2)		
Sí	111 (78,7)	103 (64,8)		
Enfermedad cardiovascular			1,963	0,161
No	114 (80,9)	138 (86,8)		
Sí	27 (19,1)	21 (13,2)		
Diabetes			8,673	0,003
No	83 (58,9)	119 (74,8)		
Sí	58 (41,1)	40 (25,2)		
Enfermedad respiratoria			0,034	0,855
No	136 (96,5)	155 (97,5)		
Sí	5 (3,5)	4 (2,5)		
Cáncer			5,410	0,020
No	126 (89,4)	153 (96,2)		
Sí	15 (10,6)	6 (3,8)		
Antecedentes de accidente cerebrovascular			122,245	< 0,001
No	30 (21,3)	135 (84,9)		
Sí	111 (78,7)	24 (15,1)		
Número de enfermedades crónicas			28,075	< 0,001
ninguna	10 (7,1)	36 (22,6)		
1 tipo	35 (24,8)	61 (38,4)		
> 1 tipo	96 (68,1)	62 (39,0)		
Si polifarmacia (≥ 5 fármacos)			11,360	0,001
No	10 (7,1)	33 (20,8)		
Sí	131 (92,9)	126 (79,2)		
Número de dientes			142,208	< 0,001
Menos de 10	82 (58,2)	5 (3,1)		
De 10 a 20	36 (25,5)	26 (16,4)		
Más de 20	23 (16,3)	128 (80,5)		
Número de dentaduras postizas M (P25, P75)	14 (4,24)	0 (0,1)	−13,120*	< 0,001
Boca seca			32,855	< 0,001
No	42 (29,8)	100 (62,9)		
Sí	99 (70,2)	59 (37,1)		
Dificultad masticatoria subjetiva			153,205	< 0,001
No	17 (12,1)	133 (83,6)		
Sí	124 (87,9)	26 (16,4)		
OHAT			130,069	< 0,001
No	113 (80,1)	23 (14,5)		
Sí	28 (19,9)	136 (85,5)		
Escala de apoyo social percibido			33,551	< 0,001
Bajo	68 (48,2)	51 (32,1)		
Medio	56 (39,7)	41 (25,8)		
Alta	17 (12,1)	67 (42,1)		
Puntuación GSEOH M (P25, P75)	46 (43,49)	55 (49,57)	−9,341*	< 0,001

### Análisis de Factor Único de Fragilidad Oral Que Afecta A Pacientes Ancianos Con Accidente Cerebrovascular

La puntuación OFI-8 de los participantes en este estudio fue de (4,87 ± 2,63), con una prevalencia de fragilidad oral del 47,0%; los resultados del análisis univariante mostraron que la edad, las condiciones laborales, el nivel educativo, el domicilio permanente, el estado civil, el pago de los gastos médicos, los antecedentes de tabaquismo, los antecedentes de hipertensión, los antecedentes de diabetes mellitus, los antecedentes de cáncer, los antecedentes de infartos cerebrales, el número de enfermedades crónicas, el uso de múltiples medicamentos, el número de dientes, el número de dentaduras postizas, la sequedad bucal, la dificultad masticatoria subjetiva, el estado de salud bucodental y la escala de apoyo social percibido y la puntuación GSEOH eran los factores asociados a la fragilidad oral en pacientes ancianos con accidente cerebrovascular, y la diferencia era estadísticamente significativa (P < 0,05), como se muestra en la Tabla 1.

### Análisis Multifactorial de La Fragilidad Oral en Pacientes Ancianos Con Accidente Cerebrovascular

La regresión logística reveló asociaciones significativas entre la fragilidad oral y varios factores, entre ellos la edad avanzada, el historial de tabaquismo, los accidentes cerebrovasculares previos, la reducción de los dientes naturales, la dificultad masticatoria subjetiva, la mala salud bucodental y el escaso apoyo social (resultados detallados en la Tabla 2). Los predictores más fuertes fueron la dificultad masticatoria subjetiva (OR = 4,743, IC del 95%: 1343–16,751) y los antecedentes de tabaquismo (OR = 3,589, IC del 95%: 1,291–9,974), seguidos de la edad (OR = 1,136, IC del 95%: 1,020–1,265).

**Tabla 2:** Resultados de la regresión logística de la fragilidad oral en pacientes ancianos con accidente cerebrovascular. este de China, 2024.

Variable	β	SE	Waldχ^2^	*P*	OR	IC
Edad	0,128	0,055	5,410	0,020	1,136	1,020–1,265
Antecedentes de tabaquismo	1,278	0,521	6,005	0,014	3,589	1,291–9,974
Antecedentes de accidente cerebrovascular	1,041	0,526	3,915	0,048	2,833	1,010–7,950
Número de dientes (menos de 10)			9,375	0,009		
10 a 20	−1,800	0,804	5,013	0,025	0,165	0,034–0,799
Más de 20	−2,899	0,947	9,372	0,002	0,055	0,009–0,352
Dificultad masticatoria subjetiva	1,557	0,644	5,845	0,016	4,743	1,343–16,751
OHAT	−1,238	0,552	5,040	0,025	0,290	0,098–0,855
Escala de apoyo social percibido (bajo)			12,226	0,002		
Medio	−2,289	0,724	9,991	0,002	0,101	0,025–0,419
Alto	−1,685	0,705	5,715	0,017	0,185	0,047–0,738

### Análisis de Regresión Logística de La Fragilidad Oral En Pacientes Ancianos Con Accidente Cerebrovascular

El AUC del modelo de regresión logística para la fragilidad oral en pacientes ancianos con accidente cerebrovascular fue de 0,943 [IC del 95% (0,916, 0,971)], como se muestra en la figura 1.

**Figura 1:** Modelo de regresión logística.

## DISCUSIÓN

### Mayor Incidencia de Fragilidad Oral En Pacientes Ancianos Con Accidente Cerebrovascular

La prevalencia entre los pacientes ancianos con accidente cerebrovascular en el este de China alcanzó el 47,0%, superando considerablemente la tasa media entre la población anciana que vive en la comunidad en China (40,2%). Cabe destacar que esta tasa también superó notablemente los registros de Japón y Finlandia. Esta disparidad probablemente se deba a: ①Vulnerabilidades específicas del accidente cerebrovascular: las deficiencias motoras/cognitivas y la disfagia comprometen de forma sinérgica la higiene bucal; ②Divergencia metodológica: los dominios funcionales de nuestra herramienta OFI-8 captan mejor las secuelas del accidente cerebrovascular que la lista de verificación centrada en indicadores clínicos de Finlandia; ③Limitaciones sanitarias regionales: el desequilibrio de recursos entre las zonas urbanas y rurales de China y los sistemas de seguro con escasa prevención retrasan las intervenciones contra la fragilidad bucal. Dado el papel fundamental de las enfermeras en el sistema sanitario chino, la formación debe hacer hincapié en la identificación de los problemas bucales relacionados con los accidentes cerebrovasculares, la incorporación de evaluaciones e intervenciones rutinarias (por ejemplo, cepillado asistido), la orientación de los pacientes y sus familias sobre el autocuidado bucal y la colaboración con equipos dentales para establecer protocolos interdisciplinarios, optimizando la coordinación de la atención para mejorar los resultados y reducir los riesgos de recurrencia de los accidentes cerebrovasculares.

### Factores Que Influyen En La Fragilidad Bucal En Pacientes Ancianos Con Accidente Cerebrovascular

El análisis de regresión logística binaria reveló que tener más de 20 dientes naturales, una mejor salud bucal (puntuación OHAT < 3) y un alto nivel de apoyo social (puntuación PSSS ≥ 61) eran factores protectores contra la fragilidad bucal en pacientes ancianos con accidente cerebrovascular, mientras que la edad avanzada, el tabaquismo, los accidentes cerebrovasculares previos y las dificultades masticatorias se asociaban de forma independiente con un mayor riesgo.

### Edad Avanzada

Los resultados de este estudio indican que el aumento de la edad se asocia con un mayor riesgo de fragilidad oral en pacientes con accidente cerebrovascular (*P* < 0,05), lo que concuerda con los resultados de estudios anteriores^(16)^. La prevalencia de la fragilidad oral aumenta con la edad, debido en gran medida a los cambios degenerativos en la función y la patología oral. Desde el punto de vista biológico, el envejecimiento reduce la regeneración de las células madre periodontales y la actividad de la fosfatasa alcalina, lo que altera el equilibrio osteogénico-osteolítico del hueso alveolar. Desde el punto de vista funcional, la disminución de la secreción salival debilita la barrera inmunitaria oral, lo que acelera la atrofia periodontal y la pérdida de integridad gingival. Desde el punto de vista patológico, los cambios degenerativos y la desregulación de la microbiota contribuyen conjuntamente a afecciones como la periodontitis y la caries dental. Estos factores crean un círculo vicioso que, en última instancia, impulsa la progresión de la fragilidad bucal^(17)^. Además, los pacientes ancianos que han sufrido un accidente cerebrovascular tienen una movilidad cognitiva y física limitada, caen en una incapacidad parcial o total y tienen una capacidad reducida para cuidarse a sí mismos, lo que conduce a una mala salud bucal, lo que a su vez aumenta el riesgo de fragilidad bucal. Los estudios han demostrado que cuanto más informados están los pacientes sobre los resultados relacionados con la mala salud bucal, más proactivos son en el mantenimiento de su salud bucal^(18)^. Para mejorar la salud bucal de los pacientes ancianos con accidente cerebrovascular, el personal de enfermería debe promover activamente la educación sobre salud bucal y proporcionar orientación sobre higiene. Reforzar la concienciación de los pacientes y fomentar hábitos de higiene bucal adecuados puede ayudar a mantener mejores resultados de salud bucal.

### Antecedentes de Tabaquismo

Los resultados de este estudio mostraron una asociación estadísticamente significativa entre los antecedentes de tabaquismo y la fragilidad bucal en pacientes ancianos con accidente cerebrovascular (*P* < 0,05). Los pacientes ancianos con accidente cerebrovascular que fuman tienen un alto riesgo de desarrollar fragilidad bucal, lo que concuerda con los resultados de estudios anteriores^(19)^. Los estudios han demostrado que el tabaquismo altera el equilibrio de la microbiota bucal, lo que favorece la disbiosis relacionada con la caries, la enfermedad periodontal y la ulceración bucal^(20)^. Además, la reducción de la diversidad microbiana bucal se asocia con problemas de salud neurológicos, cardiovasculares, respiratorios y oncológicos. El tabaquismo aumenta significativamente el riesgo de accidente cerebrovascular al provocar el endurecimiento y el estrechamiento de los vasos sanguíneos y daños en el revestimiento de los mismos, lo que reduce el suministro de sangre al cerebro. Aunque los peligros del tabaquismo son ampliamente conocidos, el 50% de los pacientes ancianos con accidente cerebrovascular de este estudio seguían fumando; por lo tanto, los profesionales de la salud deben persuadir activamente a los pacientes para que dejen de fumar y proporcionarles revisiones periódicas de salud bucal para la detección y el tratamiento tempranos de los problemas bucales causados por el tabaquismo. Al mismo tiempo, el equipo de enfermería debe impartir regularmente educación sobre el abandono del tabaquismo y la salud bucal, y proporcionar a los pacientes apoyo personalizado, como asesoramiento para dejar de fumar, intervenciones conductuales, instrucciones de higiene bucal y medicación para reducir el riesgo de fragilidad bucal y accidente cerebrovascular.

### Antecedentes de Accidente Cerebrovascular

Los resultados de este estudio mostraron que los antecedentes de infarto cerebral previo eran un factor de riesgo independiente para la fragilidad bucal en pacientes ancianos con accidente cerebrovascular (*P* < 0,05), lo que coincide con los hallazgos descritos por Hironaka et al.^(21)^. La mayoría de los pacientes con accidente cerebrovascular padecen problemas bucales en mayor o menor grado. Los accidentes cerebrovasculares pueden afectar a la función de deglución a través del daño cerebral, lo que desencadena una amplia gama de síntomas relacionados con la hipoplasia bucal. Los pacientes que han sufrido un accidente cerebrovascular suelen padecer ansiedad dental y disfagia, problemas que a menudo conducen a una fragilidad oral más grave^(22)^. La fragilidad oral no solo hace que los pacientes sean más susceptibles a las infecciones bucales, sino que también puede aumentar el riesgo de recurrencia del accidente cerebrovascular. Además, los pacientes de edad avanzada que han sufrido un accidente cerebrovascular suelen llevar dentaduras postizas debido a la pérdida de más dientes, lo que, junto con los efectos secundarios de los medicamentos para el accidente cerebrovascular, aumenta aún más el riesgo de desarrollar enfermedades bucales^(23)^. Numerosos estudios han demostrado que los adultos mayores con enfermedades crónicas como fragilidad cognitiva, enfermedad de Alzheimer, enfermedades cardiovasculares, accidente cerebrovascular y diabetes son más propensos a sufrir fragilidad oral. Los pacientes que han sufrido un accidente cerebrovascular suelen presentar disfunciones neurológicas, deterioro cognitivo, disfagia y disminución de la movilidad, lo que da lugar a una disminución de la salivación oral y la retención de alimentos, la incapacidad de limpiar la higiene bucal de manera oportuna y bajos niveles de autoeficacia en materia de salud bucal, lo que puede afectar a la función oral. Además, los estudios han demostrado que la inflamación bucal, como la pérdida de dientes y la enfermedad periodontal, puede causar infecciones bacterianas bucales que pueden promover directamente la aterosclerosis y la trombosis, lo que afecta negativamente al sistema cardiovascular^(24)^. Por lo tanto, en el caso de los pacientes de edad avanzada con antecedentes de accidente cerebrovascular, se debe reforzar el control de su función bucal, hacer hincapié en la higiene bucal y mejorar la concienciación sobre la salud bucal. Se utilizan herramientas estandarizadas en una colaboración multidisciplinaria, con el fin de evaluar la fuerza muscular oral, la presión lingual y la eficiencia masticatoria, así como la deglución compensatoria, y se lleva a cabo un entrenamiento estratégico, como la deglución múltiple y el ajuste de la posición de la cabeza, para proporcionar a los pacientes un manejo oral integral y personalizado.

### Número de Dientes, Dificultad Masticatoria Subjetiva, Estado De Salud Bucal

Los resultados de este estudio mostraron que los pacientes ancianos con accidente cerebrovascular con menos de 20 dientes, dificultades masticatorias subjetivas y mala salud bucal eran más propensos a desarrollar fragilidad bucal (*P* < 0,05), lo que concuerda con los hallazgos de Tanaka et al.^(25)^. El número de dientes, como medida de la salud general, refleja la acumulación de problemas bucales^(18)^. La pérdida de dientes puede causar directamente dificultades masticatorias, lo que no solo afecta a la ingesta nutricional y a la calidad de vida, sino que también aumenta el riesgo de muerte. Un estudio demostró que, por cada 10 dientes perdidos, el riesgo de muerte aumentaba en un 15%, un 33% y un 57%, respectivamente^(26)^. Además, un número reducido de dientes y una capacidad masticatoria disminuida se asocian con un deterioro cognitivo, mientras que aproximadamente la mitad de los pacientes con accidente cerebrovascular experimentan diversos grados de deterioro cognitivo. Las personas mayores con deterioro de la función cognitiva pueden tener un conocimiento insuficiente de la salud bucodental, lo que conduce a un menor cumplimiento de los hábitos de higiene bucodental y del autocuidado, lo que puede aumentar la magnitud de la inflamación bucodental y elevar el riesgo de fragilidad bucodental. Se ha demostrado que cuanto más saben los pacientes sobre los efectos adversos para la salud relacionados con la salud bucodental, más activos son en el mantenimiento de su salud bucodental^(27)^. La mala salud bucal incluye principalmente sequedad bucal, pérdida de dientes, enfermedad de la mucosa bucal, enfermedad periodontal o caries dental. La disminución de la secreción salival causada por la sequedad bucal aumenta el riesgo de infección por Candida albicans al debilitar los efectos inhibidores de las glicoproteínas salivales y las proteínas antimicrobianas. Por lo tanto, la insuficiencia de saliva y los malos hábitos de higiene bucal pueden causar disbiosis microbiana bucal que desencadena infecciones bucales, lo que a su vez conduce a la fragilidad bucal. Además, los pacientes de edad avanzada que han sufrido un accidente cerebrovascular y tienen menos dientes son más propensos a tener dificultades para masticar, lo que se asocia con un debilitamiento de la fuerza masticatoria y de los músculos periorales y una reducción de la eficiencia masticatoria. Esta disminución de la función masticatoria puede exacerbar el deterioro cognitivo a través de diversas vías, como la reducción de la señalización, la disminución del flujo sanguíneo cerebral y el desencadenamiento de daños crónicos por estrés oxidativo en el hipocampo y la reducción de las células vertebrales^(28)^. En resumen, los pacientes ancianos con ictus y mala salud bucal son más propensos a la fragilidad bucal, lo que no solo afecta a la función masticatoria, sino que también puede agravar el deterioro cognitivo. Por lo tanto, como profesionales de la salud, debemos reforzar la educación sobre salud bucal de los pacientes, concienciarlos sobre las consecuencias de una mala salud bucal y proporcionarles orientación profesional sobre higiene bucal. Al mismo tiempo, en el caso de los pacientes ancianos con ictus, se debe prestar especial atención a la función masticatoria y la salud bucal de los pacientes, y se debe minimizar el riesgo de fragilidad bucal y deterioro cognitivo mediante una estrategia de gestión integral que incluya entrenamiento de rehabilitación bucal y ajustes dietéticos adecuados. La salud bucal es extremadamente importante para mantener una buena salud general, y hacer hincapié en ella y mantenerla es esencial para prevenir la fragilidad bucal y mejorar la salud general de los pacientes con ictus.

### Escala De Apoyo Social Percibido

Este estudio identificó el apoyo social percibido como un factor protector independiente contra la fragilidad bucal en pacientes ancianos con ictus (*P* < 0,05), lo que concuerda con las pruebas previas de su asociación negativa con el riesgo de fragilidad bucal^(29)^. Las deficiencias motoras y cognitivas inducidas por el accidente cerebrovascular perjudican el funcionamiento social, lo que reduce las oportunidades de comunicación que provocan una disminución de la presión lingual, la capacidad masticatoria y la actividad muscular bucal^(21)^. El deterioro de la función bucal (por ejemplo, la pérdida de dientes) puede exacerbar aún más la ansiedad social y el aislamiento^(30)^, lo que fomenta la soledad y la depresión que perturban los hábitos de higiene bucal. Esto crea un ciclo «psicosocial-físico» que se refuerza a sí mismo. Las estrategias de prevención multidimensionales dirigidas por enfermeras deben integrar la evaluación de la salud mental a pie de cama, el entrenamiento cognitivo-conductual personalizado para el cumplimiento de la higiene bucal y los programas de participación social basados en la comunidad, al tiempo que se colabora con los equipos dentales para estandarizar los protocolos de cuidado bucal basados en la evidencia para esta población de alto riesgo.

## CONCLUSIÓN

La alta prevalencia de fragilidad bucal en pacientes ancianos con accidente cerebrovascular en el este de China (47,0%), que se asoció con factores relacionados con la edad avanzada, el historial de tabaquismo, los antecedentes de accidente cerebrovascular, el bajo número de dientes, la dificultad para masticar, el mal estado bucal y el bajo nivel de apoyo social, sugiere la necesidad urgente de una gestión sistemática de la salud bucal.

Las recomendaciones clave incluyen: 1) implementar exámenes OFI-8 dirigidos por enfermeras durante la hospitalización con estrategias personalizadas, como herramientas adaptativas para la hemiplejía y sustitutos de la saliva para la boca seca; 2) establecer equipos interdisciplinarios de neurología y odontología para estandarizar las vías de atención posteriores al accidente cerebrovascular. Los estudios futuros deben dar prioridad a la validación de versiones del OFI-8 adaptadas culturalmente en poblaciones infrarrepresentadas, como los residentes de zonas rurales y de residencias de ancianos, mientras que las políticas de salud pública deben exigir evaluaciones bucales en las directrices de rehabilitación y subvencionar dispositivos adaptativos para abordar las disparidades entre las zonas urbanas y rurales. Estas medidas se dirigen a las barreras universales y específicas de cada región, promoviendo una implementación global equitativa.

Este estudio tiene limitaciones: la investigación empleó un diseño transversal, que no puede determinar la causalidad entre las variables del estudio, y el muestreo de una sola región limita la generalización. Los futuros estudios longitudinales multicéntricos en diversas regiones, dirigidos especialmente a las poblaciones rurales y de residencias de ancianos, son esenciales para aclarar la progresión de la fragilidad bucal y optimizar las intervenciones.

## Data Availability

Todo el conjunto de datos que apoya los resultados de este estudio fue publicado en el propio artículo.
